# Backbone ^1^H, ^13^C, and ^15^N resonance assignments of BoMan26A, a β-mannanase of the glycoside hydrolase family 26 from the human gut bacterium *Bacteroides ovatus*

**DOI:** 10.1007/s12104-019-09879-w

**Published:** 2019-02-07

**Authors:** Sven Wernersson, Viktoria Bågenholm, Cecilia Persson, Santosh Kumar Upadhyay, Henrik Stålbrand, Mikael Akke

**Affiliations:** 1 Department of Chemistry, Biophysical Chemistry, Center for Molecular Protein Science, Lund University, Lund, Sweden; 2 Department of Chemistry, Biochemistry and Structural Biology, Center for Molecular Protein Science, Lund University, Lund, Sweden; 3 The Swedish NMR Center, University of Gothenburg, Gothenburg, Sweden

**Keywords:** Glycoside hydrolase, Polysaccharide utilization locus, β-Mannanase, TIM-barrel

## Abstract

*Bacteroides ovatus* is a member of the human gut microbiota. The importance of this microbial consortium involves the degradation of complex dietary glycans mainly conferred by glycoside hydrolases. In this study we focus on one such catabolic glycoside hydrolase from *B. ovatus*. The enzyme, termed BoMan26A, is a β-mannanase that takes part in the hydrolytic degradation of galactomannans. The crystal structure of BoMan26A has previously been determined to reveal a TIM-barrel like fold, but the relation between the protein structure and the mode of substrate processing has not yet been studied. Here we report residue-specific assignments for 95% of the 344 backbone amides of BoMan26A. The assignments form the basis for future studies of the relationship between substrate interactions and protein dynamics. In particular, the potential role of loops adjacent to glycan binding sites is of interest for such studies.

## Biological context

The β-mannanase *Bo*Man26A is a glycoside hydrolase (GH) involved in dietary glycan hydrolysis by the common human gut bacterium *Bacteroides ovatus* (Bagenholm et al. 2017). The human gut microbiota has an important influence on our health including being implicated in various diseases and drug efficiency, an example being tumor immunotherapy (Nicholson et al. 2012; Leung et al. 2016; Routy et al. 2018). Bacteroidetes is one of the dominant phyla within the gut (Eckburg et al. 2005) with members recognized for having the capacity to process and utilize complex dietary glycans (Grondin et al. 2017). The initial attack and degradation of such glycans generally involves GHs. Members of Bacteroidetes often possess gene clusters known as polysaccharide utilization loci (PULs) which in concert encode the proteins needed for the utilization of a certain polymeric glycan (Martens et al. 2009; Grondin et al. 2017). These PUL-encoded proteins include GHs, glycan-binding proteins, transporters and regulators. PUL-encoded proteins of several Bacteroides species have been studied, see recent reviews by Grondin et al. (2017) and Ndeh and Gilbert (2018).

Several hemicellulose-related PULs of the common Gram negative human gut bacterium *Bacteriodes ovatus* were previously discovered (Martens et al. 2011). One of these PULs (*Bo*ManPUL) was shown to be essential for the utilization of galactomannan (Bagenholm et al. 2017) which is a dietary β-mannan, recognized to be fermented in the human colon (Nyman et al. 1986). It has been shown that the *Bo*ManPUL encodes GHs needed for the hydrolysis of galactomannan, i.e. two β-mannanases from family GH26 (BoMan26A, BoMan26B) and a family GH36 α-galactosidase (Reddy et al. 2016; Bagenholm et al. 2017). A model for galactomannan degradation and utilization by *B. ovatus* was suggested: *Bo*Man26B is attached to the outer membrane and makes the initial attack on galactomannan and the generated oligosaccharides are processed in the periplasm involving the β-mannanase *Bo*Man26A (Bagenholm et al. 2017).

The determination of the crystal structure of the periplasmic β-mannanase *Bo*Man26A shed light on structural features that may be involved in the governance of mode of attack and product formation for this enzyme (Bagenholm et al. 2017). As expected for GH26, which is part of the large GHA-clan, *Bo*Man26A has a (β/α)_8_-barrel fold and two conserved catalytic glutamates (nucleophile and acid/base) involved in the retaining double-displacement mechanism, as investigated in detail for other GH26 β-mannanases (Bolam et al. 1996; Ducros et al. 2002). GH26 β-mannanases usually have an open active site cleft into which the β-mannan chain binds and is hydrolyzed in an endo-wise fashion (Le Nours et al. 2005; Gilbert et al. 2008). The substrate binding is conferred by subsites, each interacting with one substrate backbone monosaccharide unit. For each hydrolytic event, the β-mannosidic bond connecting the mono-sugars bound in subsites − 1 and + 1 is hydrolyzed. *Bo*Man26A is unusual in that it has two loops (loop 2: G93-S102 and loop 8: W323-S342) creating a narrow cleft beyond subsite − 2 (Bagenholm et al. 2017). The equivalent of loop 2 is also present in the exo-acting *Cellvibrio japonicus* mannobiohydrolase *Cj*Man26C (Cartmell et al. 2008). For *Cj*Man26C loop 2 is suggested to confer an exo-mode of attack because it excludes saccharide interactions beyond subsite − 2. However, for *Bo*Man26A the situation is different, since biochemical data suggest that the enzyme is able to attack substrate endo-wise and for this can bind substrate also involving a − 3 subsite (Bagenholm et al. 2017). However, current knowledge on how a saccharide would bind or be accommodated in a − 3 subsite and beyond is lacking, ligand co-crystallization has so far been unsuccessful. Potential flexibility of loop 2 and 8 could be a contributing factor to allow saccharide accommodation in a − 3 subsite and beyond. Although the B-factor of loop 8 is somewhat higher (1.5 times) than the average for crystallized *Bo*Man26A (Bagenholm et al. 2017), the potential occurrence of such flexibility has not yet been analyzed.

In order to advance the analyses of processes involved in saccharide interaction for *Bo*Man26A using NMR spectroscopy we here present backbone ^1^H, ^13^C, and ^15^N resonance assignments of *Bo*Man26A. The residue-specific assignments of *Bo*Man26A form the basis for in-depth studies of *Bo*Man26A function, including its specificity in binding various carbohydrate substrates, and the relation between conformational dynamics, ligand binding, and catalysis.

## Methods and experiments

### Protein expression and purification

A construct coding for residues 23-366 (numbered 13-356 in the current work) of *Bo*Man26A (Bagenholm et al. 2017) inserted for expression in pET-28b(+) was ordered from GenScript (Leiden, Netherlands). The sequence coding for the first 22 amino acids was omitted due the presence of a predicted site for signal peptidase I (Bagenholm et al. 2017). The sequence coding for an N-terminal His-tag and a TEV protease cleavage site (12 residues) was included, in total resulting in a construct coding for a polypeptide being 356 residues long with the *Bo*Man26A amino acid sequence starting from residue 13. The construct was transformed into One Shot™ BL21(DE3) Chemically Competent *E. coli* (Invitrogen, Thermo Fisher Scientific). The transformed cells were inoculated in 10 mL minimal media (1 mM MgSO_4_, 30 µg/mL kanamycin, 0.4 mM CaCl_2_, 1 mg/L thiamine, 1 mg/L FeCl_3_, 1 g/L NH_4_Cl, 0.5 g/L NaCl, 3 g/L KH_2_PO_4_, 6 g/L Na_2_HPO_4_ and 4 g/L glucose in H_2_O) and grown at 37 °C, 200 rpm overnight. 0.5 mL overnight culture was used to inoculate another 10 mL of minimal media (as above, except in 90% D_2_O and using [^15^N]-NH_4_Cl), which was grown over night in the same conditions. 0.5 mL of this culture was used to inoculate 20 mL of minimal media (as before, but with 100% D_2_O, [^15^N]-NH_4_Cl and [^13^C]-glucose) and grown over night in the same conditions. The cells from this culture were pelleted by centrifugation and resuspended in 1 mL supernatant. 0.5 mL of this suspension was added to 0.5 L minimal media (with 100% D_2_O, [^15^N]-NH_4_Cl and [^13^C]-glucose) and grown to an OD_600_ of about 0.7 at 37 °C, 150 rpm. When the correct OD_600_ was reached, protein expression was induced by adding isopropyl β-d-1-thiogalactopyranoside (IPTG) to a final concentration of 0.5 mM and the culture incubated for 16 h at 25 °C, 150 rpm. The cells were harvested by centrifugation and the resulting pellet stored at − 20 °C.

For purification the pellet was thawed on ice and dissolved in 35 mL lysis buffer (50 mM NaH_2_PO_4_, 0.3 M NaCl and 10 mM imidazole, pH 8) with 4 EDTA-free cOmplete protease inhibitor tablets (Roche Applied Science, Basel, Switzerland). The cells were lysed by a French pressure cell and centrifuged. The resulting supernatant was incubated at 4 °C for 1 h with 1.5 mL nickel-nitrilotriacetic acid slurry (Qiagen, Hilden, Germany) with slow head over tail rotation before being poured in to a gravity flow column, still at 4 °C. The resulting gel bed was drained and washed three times with 4 mL wash buffer (as lysis buffer, but with 20 mM imidazole) before eluting with elution buffer (as lysis buffer, but with 250 mM imidazole).

Protein concentration of the eluted fractions was measured by absorbance at 280 nm with a Nanodrop ND-1000 spectrophotometer using the theoretical extinction coefficient 89,890 M^−1^ cm^−1^ and the molecular weight 45,741 Da, calculated using the ProtParam ExPASy server (Gasteiger et al. 2005) and Biomolecular NMR tools from UC San Diego, USA: http://sopnmr.ucsd.edu/biomol-tools.htm, respectively. After assessment with SDS-PAGE (Mini-PROTEAN® TGX™ 12% precast gels, Bio-Rad) the relevant fractions were pooled, concentrated and the buffer changed to lysis buffer using 10 kDa molecular mass cutoff membrane filtration tubes (Vivaspin 20, Sartorius, Little Chalfont, UK). 8 M urea in lysis buffer was added to a final concentration of 6 M urea and incubated at room temperature with slow head over tail rotation for 1 h. This was then transferred to a 3500 Da molecular mass cutoff Spectra/Por® dialysis membrane (Spectrum Labs, Repligen, Waltham, Massachusetts, USA) and dialysed against 50 mM MES pH 6.5 at room temperature for 2 h. The dialysis solution was changed to fresh 50 mM MES pH 6.5 and the dialysis continued over night at 4 °C. The resulting protein solution was centrifuged to pellet any precipitate and the protein concentration measured using the Nanodrop instrument as described above. The activity of the enzyme was assayed using the 3,5-dinitrosalicylic acid reducing sugar assay as described previously (Stalbrand et al. 1993; Bagenholm et al. 2017) (resulting in the expected specific activity) and concentrated as described above. A final SDS-PAGE was run as above, resulting in a single band. The protein was stored in 50 mM MES pH 6.5 at 4 °C.

### NMR sample preparation

NMR samples were prepared by adding D_2_O for the field-frequency lock and transferring the protein solution to a 3 mm NMR tube. The final sample contained 0.21 mM ^2^H/^15^N/^13^C labeled BoMan26A and 10%(v/v) D_2_O in 45 mM MES pH 6.5.

#### NMR experiments

Backbone resonance assignments were carried out at 25 °C on a Bruker Avance HDIII 800 MHz spectrometer, equipped with a TCI 800S7 H-C/N-D-03 Z probe. A series of TROSY-based three-dimensional ^1^H detected spectra were acquired with deuterium decoupling using targeted acquisition (Jaravine and Orekhov 2006) and random non-uniform sampling varying between 12% and 50% completeness in the different spectra. The spectra comprised HNCO (50%), HN(CO)CA (23%), HNCA (24%), HN(CO)CACB (13%), HNCACB (22%), and HN(CA)CO (12%), where the extent of sampling resulted from the targeted acquisition protocol. Data were processed using the compressed sensing IRLS algorithm in the *mddnmr* software (Kazimierczuk and Orekhov 2011; Mayzel et al. 2014). Sequential assignment was partly achieved using the targeted acquisition approach (Jaravine and Orekhov 2006; Jaravine et al. 2008; Isaksson et al. 2013), and complemented by manual inspection of data. Automated assignment was carried out using the FLYA module of CYANA (Schmidt and Guntert 2012). The results were verified and completed manually using the CCPNmr Analysis software package (Vranken et al. 2005).

#### Assignments and data deposition

**Fig. 1:**
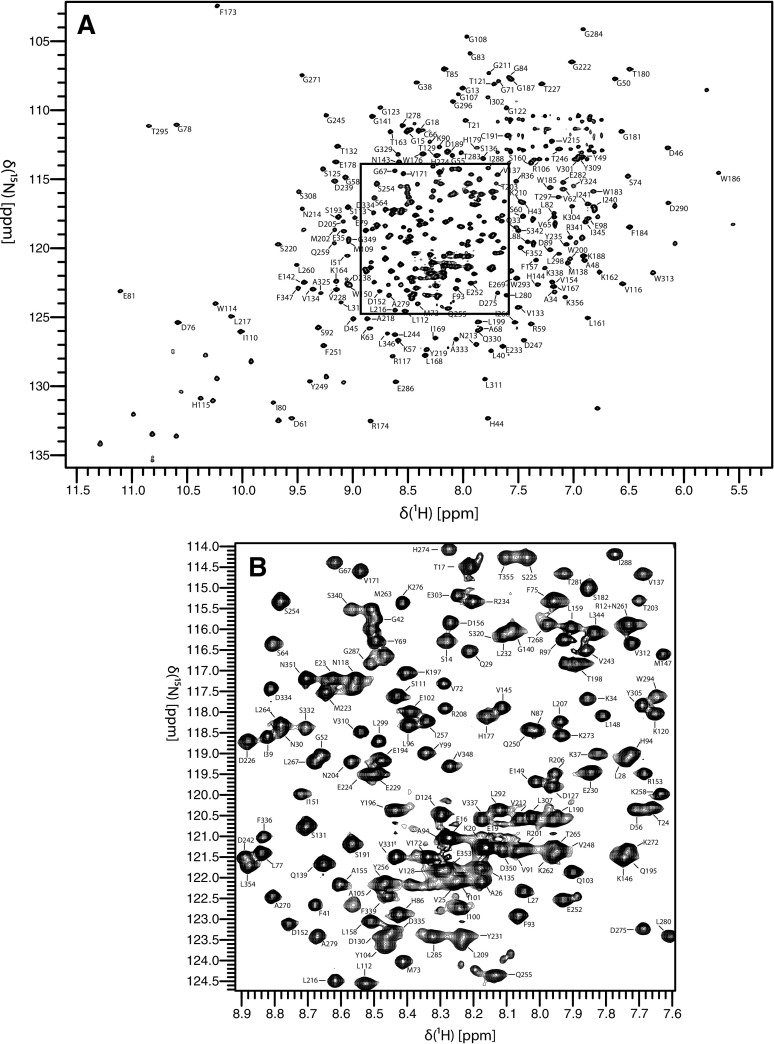
^1^H–^15^N TROSY spectrum of BoMan26A. The spectrum was acquired at a temperature of 25 °C and a static magnetic field strength of 18.8 T. **a** Overview of the full spectrum annotated with residue-specific resonance assignments. **b** Close-up view of the boxed region from **a**

**Fig. 2:**
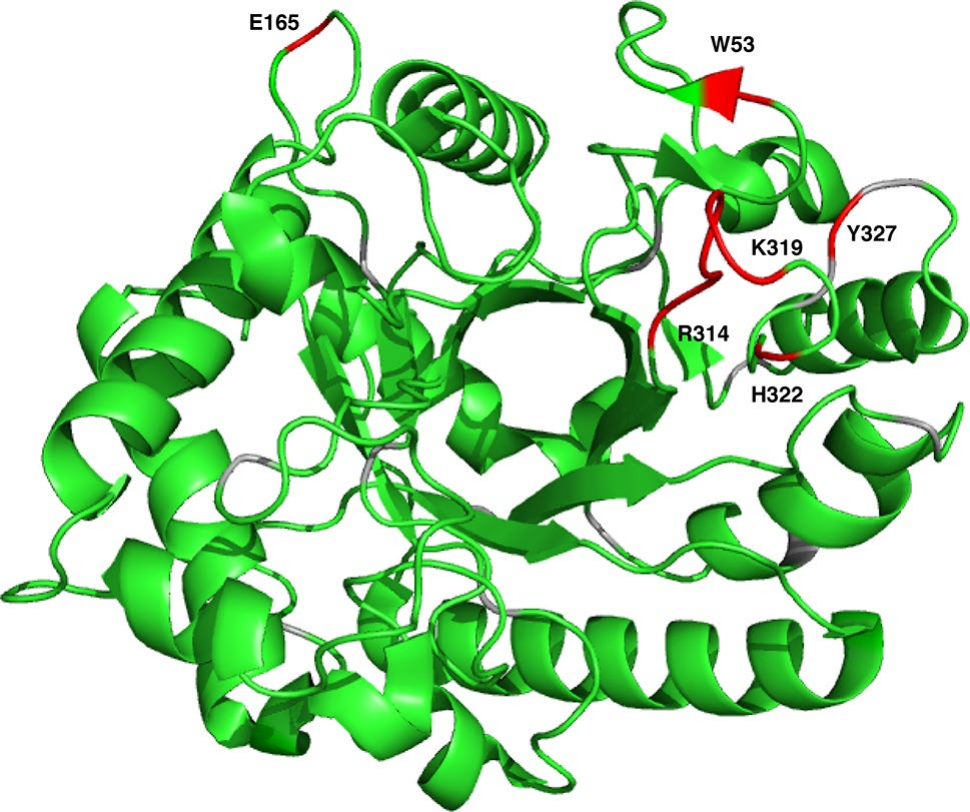
Non-assigned residues (red) mapped onto the X-ray structure of BoMan26A (PDB id 4ZXO; Bagenholm et al. 2017). The non-assigned residues are: W53, E165, R314, N315, A316, R317, E318, K319, H322, and Y327. Proline residues are colored gray

BoMan26A yields high-quality and well-resolved spectra (Fig. 1), as might be expected from its TIM-barrel like structure. The assignment procedure yielded chemical shift assignments for 95% of the H/N peaks in the TROSY spectrum. The assignment statistics are summarized in Table 1. Only 10 residues are missing assignments for all backbone chemical shifts. Eight of these residues are located close to the active site, specifically R314–K319, H322, and Y327, while W53 is located at the surface beyond loop 8 and E165 is remote from the active site (Fig. 2). Notably, the continuous stretch of missing residues, as well as H322 and Y327, are located in loop 8, which is located in the vicinity of the glycan-binding − 2 subsite (Bagenholm et al. 2017). Most likely, these residues are broadened beyond detection by exchange between alternative conformations, thereby supporting the indication that loop flexibility might be related to the mode of glycan binding and attack and thus catalytic function of BoMan26A (Bagenholm et al. 2017). The present assignments will serve as a starting point for future investigations of loop flexibility, substrate interactions, and for potentially extending the assignments by acquiring data over a range of temperatures and pH, or with different inhibitors bound.

**Table 1:** Assignment statistics

Resonance	Fraction assigned resonances^a^
N–H	315/333 Non-proline residues (95%)
C	319/348 (91%)
CA	333/348 (96%)
CB	301/315 (96%)

^a^Excluding the His-tag leader sequence (the first 9 residues)

The assigned backbone ^1^H, ^13^C, and ^15^N chemical shifts of BoMan26A have been deposited in the Biological Magnetic Resonance Bank (BMRB) under accession code 27691. This work establishes a solid basis for solution studies of BoMan26A to monitor conformational and dynamical changes induced by various natural carbohydrate ligands, as well as synthetic analogs.
